# Image-based adaptive optics for *in vivo* imaging in the hippocampus

**DOI:** 10.1038/srep42924

**Published:** 2017-02-21

**Authors:** D. Champelovier, J. Teixeira, J.-M. Conan, N. Balla, L. M. Mugnier, T. Tressard, S. Reichinnek, S. Meimon, R. Cossart, H. Rigneault, S. Monneret, A. Malvache

**Affiliations:** 1 INSERM UMR901 INMED, Institut de Neurobiologie de la Méditerranée, Aix-Marseille Université, 13273 Marseille, France; 2 Aix-Marseille Université, CNRS, Centrale Marseille, Institut Fresnel UMR 7249, 13013 Marseille, France; 3 Onera – the French Aerospace Lab, F-92322 Châtillon, France

## Abstract

Adaptive optics is a promising technique for the improvement of microscopy in tissues. A large palette of indirect and direct wavefront sensing methods has been proposed for *in vivo* imaging in experimental animal models. Application of most of these methods to complex samples suffers from either intrinsic and/or practical difficulties. Here we show a theoretically optimized wavefront correction method for inhomogeneously labeled biological samples. We demonstrate its performance at a depth of 200 μm in brain tissue within a sparsely labeled region such as the pyramidal cell layer of the hippocampus, with cells expressing GCamP6. This method is designed to be sample-independent thanks to an automatic axial locking on objects of interest through the use of an image-based metric that we designed. Using this method, we show an increase of *in vivo* imaging quality in the hippocampus.

*In vivo* imaging of neuronal calcium dynamics using two-photon microscopy is an increasingly used method of choice to study neuronal activity at microcircuit level. In the dorsal region CA1 of the hippocampus (the most optically accessible), this technique allows neuronal activity recording, in large fields of view containing hundreds of cells^1^. It has led to pioneering discoveries of multineuron dynamics including, for example fear conditioning^2^, spatial navigation^3^,^4^,^5^, epilepsy^6^ or quiet rest^7^. However, the implementation of this technique remains challenging as it requires, prior to cranial window implantation, surgery to remove the overlaying cortex, which introduces a high variability of “optical access” to the tissue. The main issues are the presence of blood from the capillaries and sometimes from small hemorrhage as well as the quality of the interface between the glass window and the brain surface. The former causes optical absorption and can be reduced by performing the surgery following water restriction to increase the viscosity of the blood^1^,^5^, while the latter causes optical aberrations. Furthermore, the densely packed layer of CA1 pyramidal neurons is located 200 μm below the glass window covering the brain; the incoming laser beam is also perturbed by light scattering and optical aberrations during the propagation within the tissue. This problem should be tackled in order to improve detection of calcium probes which is impaired by the lowered contrast of the aberrated images. Even a modest improvement in contrast should lead to the detection of neural activity that otherwise is masked by background fluorescence from brain tissue.

Optical aberrations alter the quality of beam focusing, which in turn leads to reduced spatial resolution but also to lower signal and contrast. Thus, even when objects of interest are one order of magnitude larger than the diffraction limited laser focus (e.g. neurons’ somata are 10–15 μm in diameter), the reduction of optical aberrations is critical to increasing the contrast of the fluorescence images. This improvement can be achieved using adaptive optics, a promising tool increasingly used for microscopy^8^. Adaptive optics is the process of quantifying optical aberrations through wavefront measurement and correcting them by the use of an adaptive correction element (deformable mirror DM or spatial light modulator SLM). Note that in point-scanning two-photon microscopy the correction is applied on the excitation beam alone and no correction is needed on the detection path. In such microscopes, the wavefront can either be directly measured or indirectly estimated. Direct wavefront measurement relies on introducing a wavefront sensor such as a Shack-Hartmann in the detection part of the microscope. A point source in the sample is then imaged on the sensor. Direct methods have been proposed for two-photon imaging in weakly scattering samples where auto-fluorescence signals can be used to generate a highly localized signal^9^,^10^, but more complex methods such as coherence gating^11^ or near-IR guide stars^12^ are required to avoid out-of-focus fluorescence in highly scattering samples. Indirect or sensorless wavefront estimation has the advantage of being easy-to-implement on existing systems as it relies on conventional imaging systems. Indeed, this technique, called image-based adaptive optics, relies on successive image measurements with an engineered illuminating laser beam displaying different spatial shapes either in intensity (pupil segmentation) or in phase (modal optimization).

The pupil segmentation method is based on illuminating the sample through one pupil segment at a time and measuring the image shift induced by an optical aberration consisting mainly of a local phase gradient. By comparing a reference image to images acquired with these different segments of the illuminating laser beam or beamlets, one can quantify the local phase gradient (tip/tilt) of each segment^13^,^14^. A potential issue is the loss of signal and resolution due to lower NA focusing with a truncated laser beam^15^. In the modal optimization method, one maps signal intensity or an image quality metric in phase space by applying successive wavefront deformations to the adaptive optics element. These image-based techniques, which rely on fluorescence metrics, have the main advantage that they merely require a corrective element in the illuminating laser path. However, as the aberration is indirectly inferred, the “optimized” wavefront that maximizes the quality metric is not only linked to the wavefront deformation but also to the object used for optimization. For certain spatial distributions of fluorophores, the modal optimization method may lead to a biased wavefront estimate. Thus, the quality metric and the basis used to describe the phase space as well as the method used to find the metric maximum should be carefully chosen. To address this issue we propose here an improvement of the original modal optimization scheme^16^. Note that recent so-called multiplexed methods^17^,^18^,^19^ can be seen as variations of the modal optimization scheme designed to speed up the wavefront control. Although in practice these methods may also limit the bias^18^, this has not been thoroughly studied.

The basic solution for modal optimization is the use of the mean image intensity as a metric and the Zernike modes as a basis for the phase space, the Zernike modes for tip, tilt and defocus being excluded. This intuitive approach was demonstrated *in vivo* in the mouse retina^20^. However, Zernike modes are not an orthogonal basis for our optimization problem; to be rigorous, one should iterate several times the Zernike optimization cycle to converge onto the optimal wavefront^21^. One could think of improving the optimization convergence through the use an orthogonal basis defined for the considered metric. This basis can be either theoretically computed^22^ or experimentally calibrated^16^,^23^. Such an orthogonal basis allows in principle to perform the optimization in a unique cycle, reducing the total number of measurements to a minimum of 2 images per mode plus a reference image (2 N + 1 method^23^). Note however that the very notion of orthogonal modes is only properly defined for quadratic metrics, and thus the validity of methods based on orthogonality is restricted to small aberrations for which the quality metric can be approximated by a quadratic function of the aberrations. Additionally, even in the quadratic setting, the orthogonal modes are difficult to compute theoretically because they depend on the relative geometry of {laser beam, deformable mirror, back-aperture plane}, which is not perfectly known. And the alternative experimental calibration of these modes is not an ideal solution either because it is subject to the unavoidable noise and other measurement errors. Also, as discussed in the results Section, the volumetric distribution of fluorophores induces limitations to modal optimization^24^. These limitations are only partially addressed with the construction of displacement-free modes^21^,^25^,^26^.

Even though they do not overcome all the problems, the previously proposed methods are suitable in homogeneously labeled samples^21^,^25^,^26^ or in sparsely labeled samples by selecting isolated objects^27^. However, in other cases such as *in vivo* calcium imaging in the CA1 region of hippocampus, the presence of highly inhomogeneous labeling can induce a bias in the correction (explained in the next section) that should be removed. We propose a new easy-to-implement image-based method suitable for calcium imaging in brain tissue where all these limitations are addressed. We demonstrate its performance in imaging the hippocampus both *in vitro* and *in vivo*.

## Results

As the amount of aberrations increases, the focal volume (*i.e.* the point spread function of the input beam) is distorted and enlarged, so that the maximum intensity in the focal volume decreases. It is therefore expected that the image intensity metric (mean intensity in the image) is maximum when there is no aberration in the system. However, when considering a heterogeneously labeled 3D sample, this is not true anymore. For instance, if a high fluorophore concentration is in the vicinity of the focal plane, an iterative modal optimization of the aberration will lead to adding tip/tilt and defocus to shift the focal plane onto this location. This is why one generally forbids tip/tilt and defocus to avoid changing the actual location of the scan. Still, even with these precautions, the optimization will lead to “stretching” the point spread function so that it reaches the location of the bright fluorophore source. In other words, for some fluorophore distributions, an increase of aberrations can increase the intensity metric value while worsening the quality of the laser beam focus and thus degrading the overall resolution and contrast of the data. In such undesirable situations, the wavefront estimation is thus biased and is called “sample dependent”. Note that this bias effect has been shown in third harmonic generation (THG) microscopy, where increasing the amount of aberrations could increase the THG mean intensity for some specific sample geometry^22^,^24^. This effect is also expected in two-photon excitation fluorescence microscopy^24^,^27^.

**Figure 1:**
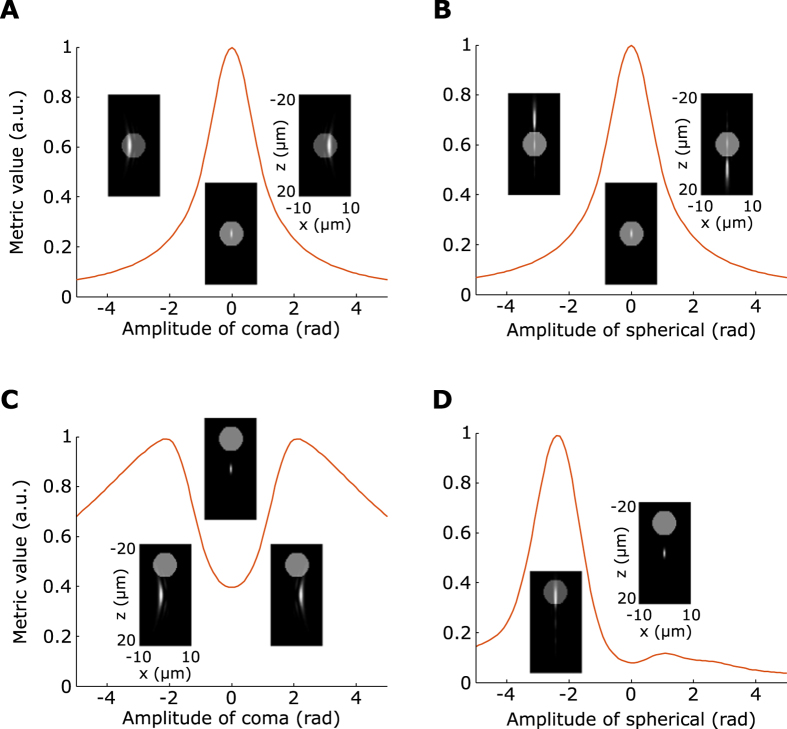
Sample-dependence of image-based correction methods. Simulated variation of the mean image intensity metric with respect to aberration amplitude with a 10 μm fluorescent bead in focus (**A**,**B**) and 12 *μm* out-of-focus (**C**,**D**) for coma (**A**,**C**) and spherical aberration (**B**,**D**). Insets: schematics of the 2D axial profile (xz) of the point spread function and the fluorescent bead for different aberration amplitudes: −2.1, 0, 2.1 rad of coma (**A**,**C**); −2.3, 0, 2.3 rad of spherical aberration (**B**) and −2.3, 0 rad of spherical aberration (**D**). The focused beam propagates along the Z axis.

To quantify this phenomenon, we have developed a numerical tool simulating a two-photon laser scanning microscope. The two-photon excitation laser focal volume is calculated using a diffraction model, and then convolved with an object (see methods for more details). Figure 1 shows the evolution of the mean image intensity metric *M*_1_ for a given transverse scan for different amplitudes of coma and spherical aberration when imaging a 10 μm fluorophore bead in-focus and out-of-focus. As expected, when focusing on the bead (Fig. 1A,B), the maximum is obtained in the absence of aberrations. However, we observe that, if the bead is 12 μm out-of-focus (Fig. 1C,D) the metric maxima are obtained for a large amount of aberration. Increasing the amount of coma causes an elongation/distortion of the focal volume and increases its interaction with the bead (Fig. 1C). For the spherical aberration, increasing the amount of aberrations causes a distortion and a displacement of the focal volume also resulting in a better interaction with the bead (Fig. 1D). This example on an isolated object can be generalized to any heterogeneously labeled sample. The modal optimization method may therefore lead to a biased estimation of the aberration that is linked to the volumetric distribution of contrast agent in the object.

Several groups have attempted to overcome this so-called sample dependency by the use of a basis of displacement-free modes^21^,^25^,^26^. However, with these displacement-free modes, the issue concerning the elongation/distortion of the focal volume remains (for example, see the effect induced by the coma aberrations on Fig. 1), as already noted in ref. 27. To overcome the sample dependency problem one may also think of using a different metric. We however show that sharpness type metrics do not solve the elongation/distortion issue (see Fig. S1). A metric based not on a single image but on the volume intensity is a solution^24^, but it requires the acquisition of several z-stacks for each optimization mode. Such a time consuming procedure is not applicable to *in vivo* imaging.

The inhomogeneous labeling of biological media thus results in the sample dependency problem with the current modal optimizations. In contrast, we wish here to exploit our prior knowledge on the object characteristics, in our case the presence of 10 *μm* size neurons, in order to access wavefront measurements that are not biased by the detailed 3D structure of the sample. Instead of constraining the optimization to “stay away” from a strong fluorophore concentration such as neuron somata, we take the opposite strategy of “locking” on it, and optimizing the aberrations around it. In this sense, we make use of the strongest light source in the vicinity, instead of fighting its influence on the optimization.

We thus designed a procedure called “axially-locked modal optimization”, which consists in performing the two following steps:

1. Find a local maximum of an intensity related metric in the axial (z) dimension.
2. At this depth, optimize the metric iteratively for each Zernike mode including defocus.

Contrary to previous works, our new strategy therefore allows controlled shifts in depth (defocus)- the first step operates a coarse focus while fine tuning of defocus is performed in the second step. Our approach is opposite to the displacement-free modal optimization principle which implements optimization at a fixed depth and suffers from the inherent limitation mentioned earlier. In contrast, our strategy of locking focuses on bright structures to eliminate the risk of introducing aberrations during the optimization process which helps us to image structures of our interest with improved contrast.

**Figure 2:**
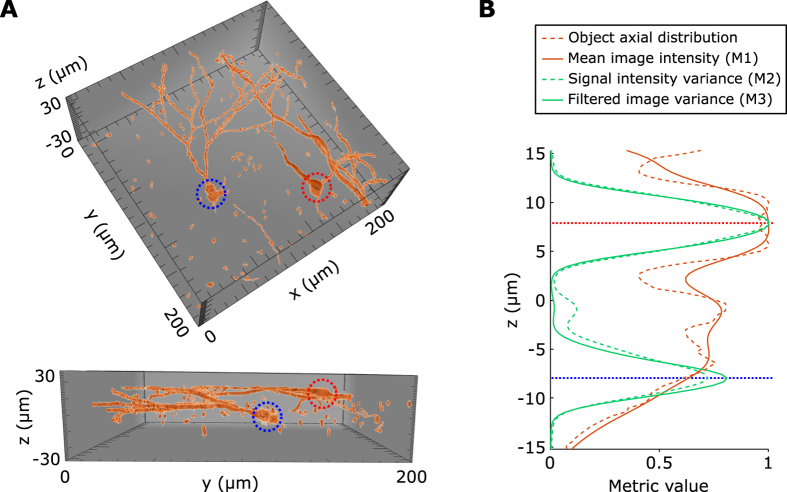
Simulated axial variation of different metrics. (**A**) 3D views of the neuron model: two neurons somata (dotted circles) and several dendrites built using fixed hippocampal slices of GFP-expressing neurons. (**B**) Axial variation of the three considered metrics, the somata positions are indicated by the blue and red dotted line respectively.

The first step of our axially-locked modal optimization method requires a specific intensity-related metric that presents local maxima around the region of interest. In our case, it has to be able to locate the depth of labeled somata. To compute such a metric, we used a 3D digitized sample representing a brain slab (Fig. 2A) including two somata (centered at *z* = −8 *μm* and *z* = 8 *μm*, respectively) as well as dendrites, built using confocal images of GFP-expressing neurons in fixed hippocampal slices. Figure 2B shows the evolution of different intensity-related metrics as a function of depth, on simulated transverse scans of the 3D model. We observe that the mean image intensity *M*_1_ does not allow determining the depth of the somata; it does not distinguish layers with many small structures (*e.g.* around *z* = 0 *μm*) from layers with a well-defined soma (around *z* = −8 *μm*). One can actually show that the mean image intensity does not depend on the transverse (xy) structure of the object and only depends on its mean axial distribution (see Suppl. Mat.) which forbids object-locking. We therefore need to investigate other intensity-related metrics.

We can observe that the image intensity variance (also known as sharpness metric) *M*_2_ (see Suppl. Mat.) displays two local maxima close to the positions of the two somata (Fig. 2B). However, it also displays a local maximum in between the somata due to the presence of dendrites. We thus defined a new metric *M*_3_ which consists in filtering out low and high spatial frequencies of the image, to increase the contrast between somata and dendrites, before calculating the intensity variance (we call this metric “filtered image sharpness metric”, see Suppl. Mat.). The exact filtering parameters are defined such that the objects of interest (i.e. neurons somata) are highlighted. As displayed in Fig. 2B, *M*_3_ improves the precision in localization. Thus, step 1 of our method consists in computing *M*_3_ values as a function of depth, and then setting the imaging plane at the depth that maximizes *M*_3_.

**Figure 3:**
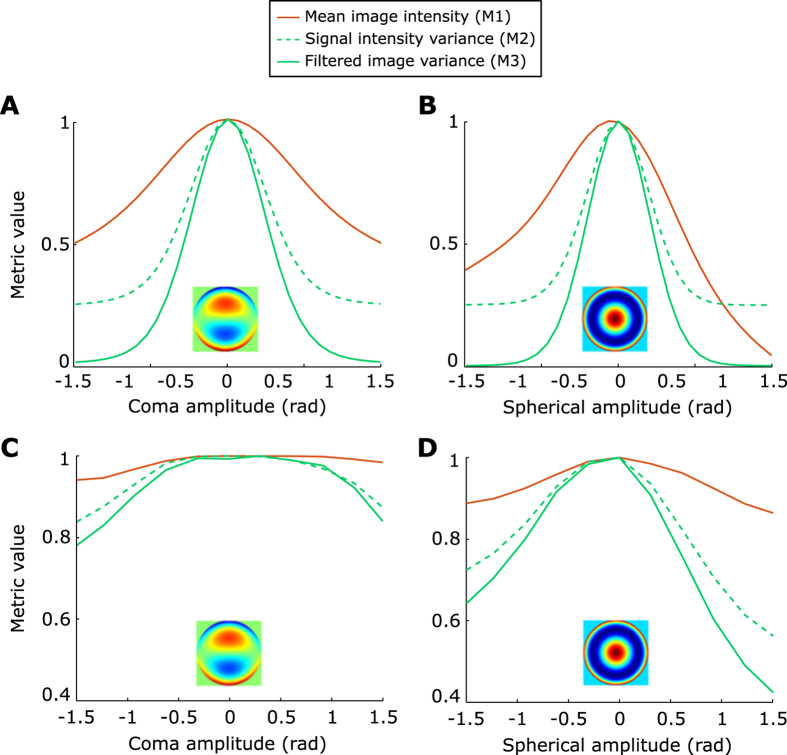
Sensitivity of the different metrics to aberrations. Variation of the three considered metrics with respect to coma (**A**,**C**) and spherical aberration (**B**,**D**), using simulated transverse scans, at *z* = −8 *μm*, on the 3D reconstructed neurons with detection noise (**A**,**B**) and using experimental transverse scans of GAD67KI-GFP hippocampal slices in a densely packed region 100 μm deep (**C**,**D**). Insets represent the phase profiles of coma and spherical aberrations.

In the next step of optimizing aberration corrections (step 2), the relative sensitivity of the different intensity related metrics to aberrations can be compared using our simulation with the digitized sample (Fig. 3A,B) and experimental results obtained in hippocampal slices GAD67KI-GFP of mice in which 10–20% of the neurons, the GABAergic ones, express GFP (Fig. 3C,D). We observed that the mean signal intensity *M*_1_ is less sensitive to aberrations than metrics *M*_2_ and *M*_3_. We also observed that *M*_3_ is the most sensitive, both in simulations and experiments. To conclude, the filtered image sharpness metric (*M*_3_) appears well-suited for both steps 1 and 2 of the axially-locked modal optimization method, in samples that contain objects well defined in shape.

In order to test our axially-locked modal optimization method, we built a custom-made point-scanning two-photon microscope (see methods and Fig. S1) similar to the commercial one we used for hippocampal *in vivo* large scale calcium imaging^5^,^6^,^7^. Wavefront control was performed using a deformable mirror (IrisAO, PTT111-5) placed into the illuminating laser path. We applied the following procedure in fixed hippocampal slices of GAD67KI-GFP mice and in the hippocampus of living mice.

*Experimental procedure for axially-locked modal optimization*:

1. Acquisition of a 40 μm z-stack around the field of view (FOV) of interest using a default wavefront (setup correction).
2. Calculation of *M*_3_ (*z*).
3. Setting the imaging plane at the depth z0 that maximizes *M*_3_.
4. Explore all the N Zernike modes amplitudes (4N + 1, including defocus) around the current wavefront from −1.5 to 1.5 rad and compute *M*_3_. For each mode, store value of coefficient that maximizes *M*_3_.
5. Update the wavefront by using the coefficients computed in step 4.
6. Repeat steps 4 and 5 twice (i.e. 3 iterations in total).
7. Image the FOV with the final correction and with the initial wavefront for comparison.

For the method to be robust and efficient, we applied 4 different amplitudes to each Zernike mode (4N + 1 method) with 3 iterations to account for the couplings between the Zernike modes^21^. As explained in the introduction, couplings are difficult to avoid in practice hence our choice to rather iterate on a standard Zernike basis. The initial setup correction was first defined using the above procedure on calibration samples (1 μm fluorescent beads), using the *M*_1_ metric.

### *In vitro* use of the method

**Figure 4:**
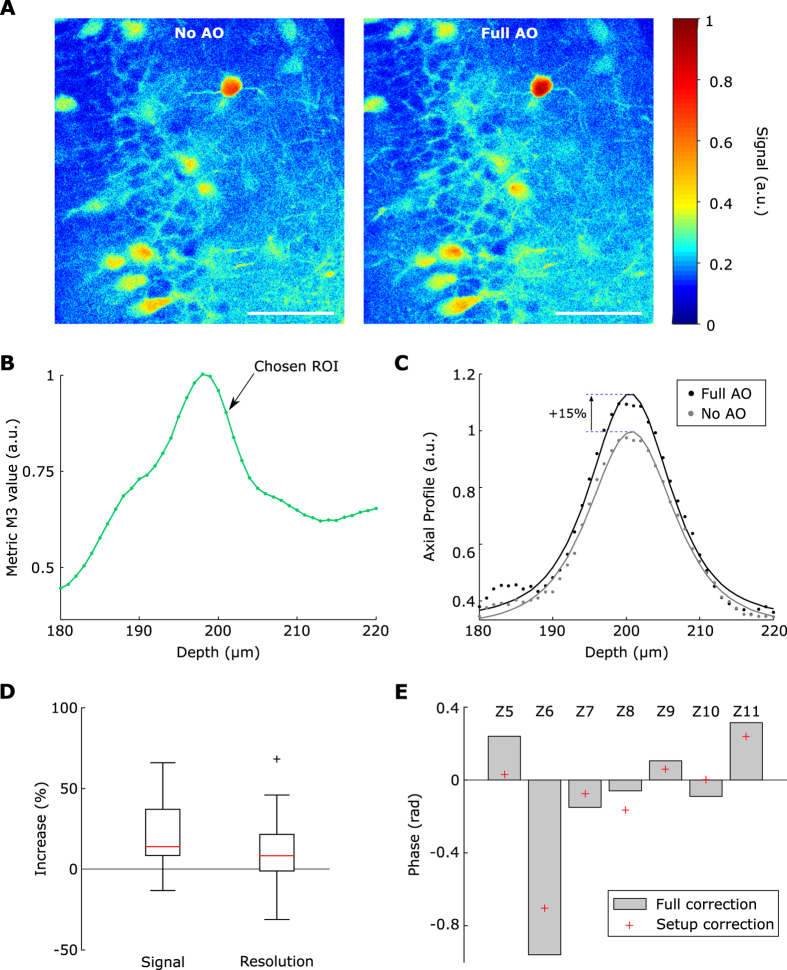
Axially-locked method in hippocampal slices. (**A**) Example ROI, 200 μm deep in a GAD67KI-GFP hippocampal slice, without and with wavefront correction (No AO and Full AO), scale bar: 100 μm. (**B**) Axial variation of the metric *M*_3_ in the same slice, the maximum of the metric was at 198 μm. (**C**) Axial profiles of a representative neuron without and with wavefront correction (No AO and Full AO) and their respective fit. (**D**) Signal and resolution increase with wavefront correction, calculated using the axial fit of n = 36 somata; boxplots showing median (red lines) and inter-quartile range (IQR, rectangle), whiskers and outliers are calculated with a 1.5*IQR threshold. (**E**) Median amplitude of the correction (n = 5 FOVs) on each considered Zernike modes for setup correction and full (setup + sample) correction, Zernike modes are indexed following the A176988 sequence.

We tested this method on fixed hippocampal slices (see methods) from GAD67KI-GFP mice at depths ranging from 180 μm to 200 μm. We focused on 400 × 400 μm^2^ fields-of-view (FOVs) with densely packed neurons (the pyramidal cell layer of CA1 and CA3 or the granular cell layer of the dentate gyrus). An example of FOV is displayed in Fig. 4A. We then applied our method with N = 8 Zernike modes, for 5 different FOVs, 200 μm deep (n = 3 slices). Photobleaching was compensated for by taking into account the exponential decay of intensity across all images. A local maximum of *M*_3_ was typically found within 10 μm around the chosen ROI (Fig. 4B). To estimate the performance, 3D images were recorded (512 × 512 × 40px^3^, FOV 400 × 400 × 40 μm^3^) with and without correction and the axial profile of selected somata was fitted (Fig. 4C). The axial profile was calculated by averaging the signal on a 12 μm-diameter disk centered on the soma. The function used for the fit was the square of the Gaussian beam axial profile which is a reasonable approximation of the aberrated two-photon PSF convolved with a neuron (see methods). For a given neuron, the signal was defined as the difference between the maximum of the fit and its minimum (background signal), the axial resolution was defined as the Full Width at Quarter Maximum of the fit. We obtained a signal increase of 14% and an axial resolution improvement of 8.5% (median value, n = 36 somata, Fig. 4D). The corrected wavefront mainly contained astigmatism (Z5 and Z6) and spherical aberration (Z11) (Fig. 4E). It closely resembled the one obtained for setup correction only (Fig. 4E), which explains why the signal and resolution improvements with setup correction and sample correction were not statistically different. Thus, at depths of 200 μm in fixed brain slices, the sample induced wavefront aberrations on the laser were minimal. However, this result shows that the axially-locked modal optimization can reliably be used in brain tissue with fluorescently labelled neurons.

### *In vivo* use of the method

**Figure 5:**
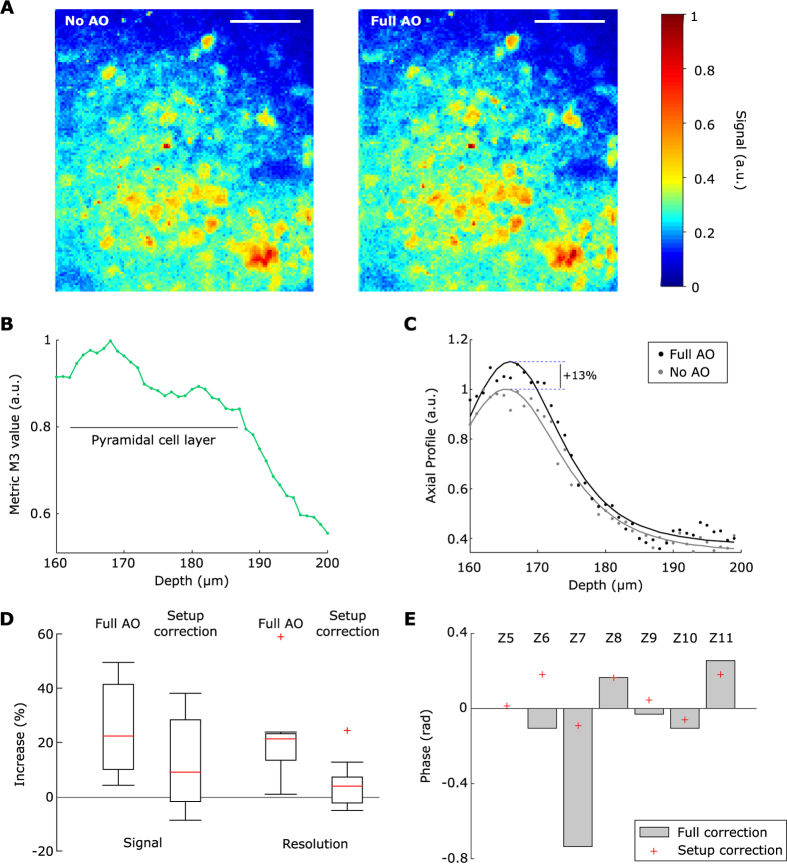
Axially-locked method in living mouse. (**A**) Example ROI, 175 μm deep in the CA1 hippocampal region of a living mouse, without and with wavefront correction (No AO and Full AO), scale bar: 100 μm. (**B**) Axial variation of the metric *M*_3_ in the same slice, the maximum of the metric was at 168 μm, the pyramidal cell layer spanned from 162 to 187 μm. (**C**) Axial profiles of a representative neuron without and with wavefront correction (No AO and Full AO) and their respective fit. (**D**) Signal and resolution increase with wavefront correction and setup correction, calculated using the axial fit of n = 11 somata; boxplots showing median (red lines) and inter-quartile range (IQR, rectangle), whiskers and outliers are calculated with a 1.5*IQR threshold. (**E**) Amplitude of the correction on each considered Zernike modes for setup correction and full (setup + sample) correction, Zernike modes are indexed following the A176988 sequence.

We finally used this correction method for *in vivo* imaging of the CA1 pyramidal cell layer through a previously developed chronic cranial window implanted above the hippocampus after the removal of the overlaying cortex^1^,^5^. The expression of the calcium indicator GCamP6f was virally-induced (see methods). Aberration correction was performed in anesthetized mice on a 400 × 400 μm^2^ FOV (Fig. 5A), no photobleaching was observed. Doing so, a local maximum of the metric *M*_3_ was found within the pyramidal cell layer (Fig. 5B). The axial profile of 11 somata located within this layer were calculated (Fig. 5C), and a signal enhancement of 21% and an axial resolution enhancement of 21% (median values, Fig. 5D) were obtained. This enhancement of image quality was significantly higher than just setup correction, which led to a 6% signal and 4% resolution enhancement (median values, Wilcoxon rank-sum test, Fig. 5D). Most of the correction originated from the horizontal coma (Z6 in Fig. 5E) which can be explained by the curved shape of the different layers of CA1.

## Discussion

We propose a new image-based adaptive optics method that is designed for imaging heterogeneously labeled scattering samples such as the pyramidal cell layer of the hippocampus. It relies on a specific metric which consists in filtering out low and high spatial frequencies of the image before calculating the intensity variance. Thanks to this ‘filtered image sharpness metric’, we exploit the stereotyped motif of the labeling (e.g. the neurons) to lock on the optimal layer depth before performing aberration estimation. Most importantly, we show that this axially-locked modal optimization is well-suited to enhance the image quality of CA1 pyramidal cells in *in vivo* large scale imaging of neural activity, where previously reported modal optimization methods are not optimal because of the highly inhomogeneous labeling.

Compared to direct measurement methods, our method is easy-to-implement as it only requires adding a wavefront correction device in a standard microscope to manipulate the canonical Zernike modes. Furthermore, it can readily be applied on biologically relevant samples such as GCamP expressing neuron because it does not require isolated objects thanks to its low sensitivity to inhomogeneous labeling. Thus, this technique is suitable for all kinds of applications which involve fluorescence imaging in deep tissues.

As far as acquisition time is concerned, 3 iterations of the 4 N + 1 scans of 8 Zernike modes requires 99 images which would take160 s for 512 × 512px^2^ image size (*in vitro*) and 10 s for 128 × 128px^2^ image size (*in vivo*). However, if one wants to apply this method to non-anesthetized mice, the variability in fluorescence related to spontaneous neuronal activity will have to be taken into account. One can take advantage of the sparse spontaneous activity - by performing multiple measurements and using quantification metrics which are robust to outliers, it should be possible to obtain a reliable correction.

Although a relatively mild improvement in signal and resolution (~20%) could be obtained, it should be sufficient to increase the probability of detecting small changes in fluorescence such as neurons firing sparsely in time. For example, in the case of hippocampal reactivations where assemblies of neurons fire a few action potentials^7^, the image quality was critical to reliably detect these events: typically 50% of detected events had calcium signals less than 20% above noise level. Signal enhancement could also be used to facilitate imaging the hippocampal dendritic activity *in vivo*, which also displays weak fluorescence changes^3^. We thus expect that applying axially-locked modal optimization will significantly improve the data quality of *in vivo* calcium imaging in the hippocampus and in other regions of the brain.

## Methods

All protocols were performed under the guidelines of the French National Ethics Committee for Sciences and Health report on “Ethical Principles for Animal Experimentation” in agreement with the European Community Directive 86/609/EEC. The experimental protocols were approved by the French National Ethics Committee under agreement #01413.03.

### Simulated imaging

A simulated two-photon laser scanning microscope based on a diffraction model for 3D PSF in the presence of aberrations was used to compare intensity-related metrics (*M*_*1*_, *M*_*2*_ and *M*_*3*_) and the different strategies (standard and axially-locked). The parameters of the microscope were chosen to match the experimental setup. The 3D PSF is convolved by different objects (for example, beads and 3D model of brain slice, possibly adding detection noise) to obtain the z-stacks and the different metrics (see supplementary materials for more details).

### Two-photon Laser Scanning AO microscope

The system is a wavefront corrected two-photon imaging system (see Fig. S2). The NIR excitation beam (920 nm) from a pulsed laser (Chameleon Ultra II, Coherent Inc.) goes through a power control system consisting of a half-wave plate and a polarized beam splitter. Then it is expanded and collimated by a pair of lenses (L1 = −50 mm and L2 = 150 mm). The collimated NIR beam slightly overfills the aperture of a deformable mirror (DM, PTT111-5, IrisAO, Inc.) and is reflected with a shaped wavefront. The DM is imaged onto the two scanning galvanometric mirrors (6200 H, Cambridge Technology, Novanta, Inc.) by two pairs of relay lenses (focal lengths: L3 = 750 mm, L4 = 1000 mm, L5 = 400 mm and L6 = 400 mm) and then to the back pupil of the objective (16X/0.8, Plan Fluorite Physiology, Nikon Instruments Europe B.V.) by another pair of relay lenses (focal lengths: L7 = 50 mm and F8 = 200 mm). The back-aperture of the objective was just filled with the image of DM in order to reach maximum numerical aperture. The objective focuses the beam into the sample and collects the excited fluorescence. Fluorescence is reflected by a dichroic mirror D (FF757-Di01, Semrock, Inc.) placed immediately after the objective and guided by a pair of lenses (focal length: L9 = 150 mm and L10 = 50 mm) onto a photomultiplier tube PMT (H7422P-40, Hamamatsu Photonics K.K.). Two short pass filters (2xSPF, FESH750, Thorlabs, Inc.) are added between the two lenses and a GFP emission filter (GFPEF, MF525-39, Thorlabs, Inc.) is added in front of the detector to clean the fluorescent signal from any unwanted light.

### *In vitro* preparation

Adult GAD67-green fluorescent protein-knock-in (GAD67-GFP-KI) mice^28^ were anesthetized with a ketamine (250 mg/kg) and xylazine (25 mg/kg) solution (i.p.) and transcardially perfused with 4% paraformaldehyde in PBS (1 ml/g). Brains were postfixed overnight, washed in PBS, and kept at −20 °C for long-term storage. 600 μm thick coronal brain sections were prepared and processed.

### *In vivo* preparation

Mice were handled before recording sessions to limit head restraint-associated stress and experiments were performed during the dark cycle. The analgesic (Buprenorphine, 0.1 mg/kg) was administrated before any surgery. Viral infection was previously described^5^, however the virus stock solution was diluted by 1:5 (D-PBS Sigma-Aldrich) and the resultant solution was injected 2 times 200 nl of AAV2/1.Syn.GCamP6f.WPRE.SV40 (Penn Vector Core) (AP −2.0/2.5, ML 1.6/2.1 and DV −1.3). The head-fixation bar (custom-made aluminum bar) was firmly secured with dental cement (GripCement, SuperBond, Sun Medical). Behavioral handling and imaging procedures were optimized and performed similarly as described previously^5^.

### Axial profile fit

In order to fit the axial profile of neurons, we used the square of the Gaussian beam axial profile


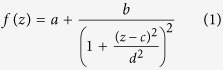


where *a, b, c* and *d* are adjustable parameters. *a* represents the background level, *b* is the maximal intensity of the neuron, *c* its depth and *d* its the Full Width at Quarter Maximum.

### Data availability

Supporting data is accessible on Figshare: https://dx.doi.org/10.6084/m9.figshare.4012737.v1.

## Additional Information

**How to cite this article:** Champelovier, D. *et al*. Image-based adaptive optics for *in vivo* imaging in the hippocampus. *Sci. Rep.* **7**, 42924; doi: 10.1038/srep42924 (2017).

**Publisher's note:** Springer Nature remains neutral with regard to jurisdictional claims in published maps and institutional affiliations.

## Supplementary Material

Supplementary Material
