# Supplementary material for: Image-based adaptive optics for in vivo imaging in the hippocampus
Source: Sci Rep. 2017 Feb 21;7:42924. doi: 10.1038/srep42924 (PMC5318884; doi:10.1038/srep42924)
Supplement: Supplementary Material [file srep42924-s1.pdf]

Image-based adaptive optics for in vivo imaging in the hippocampus

D. Champelovier[#1,2], J. Teixeira[#3], J-M. Conan[3], N. Balla[2], L. Mugnier[3], T. Tressard[1], S. Reichinnek[1], S. Meimon[3], R. Cossart[1], H. Rigneault[2], S. Monneret[2], A. Malvache*[1,2]

[1] INMED, Aix-Marseille Univ, INSERM, Marseille, France
[2] Aix-Marseille Univ, CNRS, Centrale Marseille, Institut Fresnel UMR 7249, 13013 Marseille, France
[3] Onera – the French Aerospace Lab, F-92322 Châtillon, France
[#] These authors contributed equally to the work
*Correspondence: arnaud.malvache@inserm.fr

Supplementary material

Derivation of the two-photon focal volume in the presence of aberrations

The single-photon 3D point spread function (3DPSF) is described by:

$$h_{\boldsymbol{a}}(x,y,z) = \left| FT_{2D}\left[P(r_x,r_y)\, G(r_x,r_y)\, \exp\left(i\varphi(r_x,r_y) + i a_4(z)\, Z_4(r_x,r_y)\right)\right]\right|^2 \quad (1)$$

where $P(r_x,r_y)$ represents the back-aperture pupil support, $G(r_x,r_y)$ the Gaussian profile of the laser, $\varphi(r_x,r_y) = \sum_{i=1}^{n} a_i\, Z_i(r_x,r_y)$ represents the wavefront phase and Z_i the i-th Zernike mode. Here, Z_4 correspond to defocus and $a_4(z)$ is an artificial defocus that allows to compute the PSF for the plane z. The aberrations are represented by the vector $\boldsymbol{a} = (a_1, \dots, a_n)$.

The two-photon 3D PSF, also known as the two-photon focal volume, is $h_{\boldsymbol{a}}^2$.

Definition of the metrics

For two-photon microscopy, the image intensity is given by the 3D-convolution of the 3D PSF $h_{\boldsymbol{a}}^2$ and η, the fluorescence efficiency in the sample volume, called hereafter the “object”.:

$$I(x,y,z) = h_{\boldsymbol{a}}^2(x,y,z) \star_{3D} \eta(x,y,z) \quad (2)$$

The modal sensorless approach implies the optimization of an image-based metric computed on a transverse scan. One common metric is the total image intensity (Débarre 2009):

$$M_1(\boldsymbol{a}; z_0) = \int I(x,y,z_0)\, dx\, dy \quad (3)$$

Another common metric is the image sharpness metric (Fienup 2003). Metrics based on image sharpness are also widely used in different image processing applications (Kubby 2013, Sulai 2014 and Wahl 2015). A common definition of image sharpness metric is the second order moment of the image. Other authors rather use the image intensity variance (Girkin 2013). We prefer here the latter choice that allows to be insensitive to constant background. The image variance metric reads:

$$M_2(\boldsymbol{a}; z_0) = \frac{1}{S}\int I^2(x, y, z_0)\, dx\, dy - \frac{1}{S^2}\left(\int I(x, y, z_0)\, dx\, dy\right)^2 \quad (4)$$

S is the surface of the FOV. A pre-filtering can be applied to the image to enhance some particular structures. In our study, we considered a filter F that enhances structures with a given characteristic size. The pre-filtered image for a transverse scan at z_0 can be represented by the equation:

$$I_F(x, y, z_0) = I(x, y, z_0) \star_{2D} F(x, y) \quad (5)$$

For 10µm-size objects like neurons, F is an annular Gaussian centered at frequency 0.1µm^{-1} with a half width at 1/e of 0.1µm^{-1}.

Then, we can now define another metric, called hereafter *pre-filtered image variance*, as:

$$M_3(\boldsymbol{a}; z_0) = \frac{1}{S}\int I_F^2(x, y, z_0)\, dx\, dy - \frac{1}{S^2}\left(\int I_F(x, y, z_0)\, dx\, dy\right)^2 \quad (6)$$

Derivation of the transverse structure independence of M1

The fluorescence intensity for a point (x_0, y_0) of a transverse scan at a depth z_0:

$$I(x_0, y_0; z_0) = \iiint h_{\boldsymbol{a}}^2(x' - x_0, y' - y_0; z - z_0) \cdot \eta(x', y'; z)\, dx'\, dy'\, dz \quad (7)$$

$$I(x_0, y_0; z_0) = \int \left(h_{\boldsymbol{a}}^2(x_0, y_0; z - z_0) \star_{2D} \eta(x_0, y_0; z)\right) dz \quad (8)$$

The total image intensity of a XY scan at z_0 is given by:

$$M_1(\boldsymbol{a}; z_0) = \iint I(x_0, y_0; z_0)\, dx_0\, dy_0 = \iint \int \left(h_{\boldsymbol{a}}^2(x_0, y_0; z - z_0) \star_{2D} \eta(x_0, y_0; z)\right) dz\, dx_0\, dy_0 \quad (9)$$

We recall that the integral of a function is equal to is value at 0, in the frequency domain, of its Fourier transform: $\int_{\mathbb{R}^n} g(x_1, \cdots, x_n)\, dx_1 \cdots dx_n = FT[g](0, \cdots, 0)$.

Applying this property to the 2D Fourier transform with respect to transverse coordinates ($FT_{2D}[\cdot]$) we obtain:

$$M_1(\boldsymbol{a}; z_0) = \int FT_{2D}\left[h_{\boldsymbol{a}, z-z_0}^2 \star_{2D} \eta_z\right](0,0)\, dz = \int \left[FT_{2D}\left[h_{\boldsymbol{a}, z-z_0}^2\right](0,0) \cdot FT_{2D}[\eta_z](0,0)\right] dz \quad (10)$$

$$M_1(\boldsymbol{a}; z_0) = \int \left[\iint h_{\boldsymbol{a}}^2(x_0, y_0; z - z_0)\, dx_0\, dy_0 \cdot \iint \eta(x_0, y_0; z) dx_0\, dy_0\right] dz \quad (11)$$

$$M_1(\boldsymbol{a}; z_0) = \int \overline{h_{\boldsymbol{a}}^2}(z - z_0) \cdot \bar{\eta}(z)\, dz \quad (12)$$

When, $\overline{h_a^2}(z) = \int h_a^2(x, y, z)\, dx\, dy$ represents the mean axial distribution of the two-photon focal volume and $\bar{\eta}(z) = \int \eta(x, y, z)\, dx\, dy$ represents the mean axial distribution of the object.

Figure S1: Changing the intensity related metric does not remove sample-dependency

Image quality metrics (no detection noise) as function of aberrations Z7 and Z11 (upper and lower graphics respectively) with a $10\,\mu m$ bead in-focus and $12\,\mu m$ out-of-focus (left and right respectively). The sample dependency can be observed for all metrics.

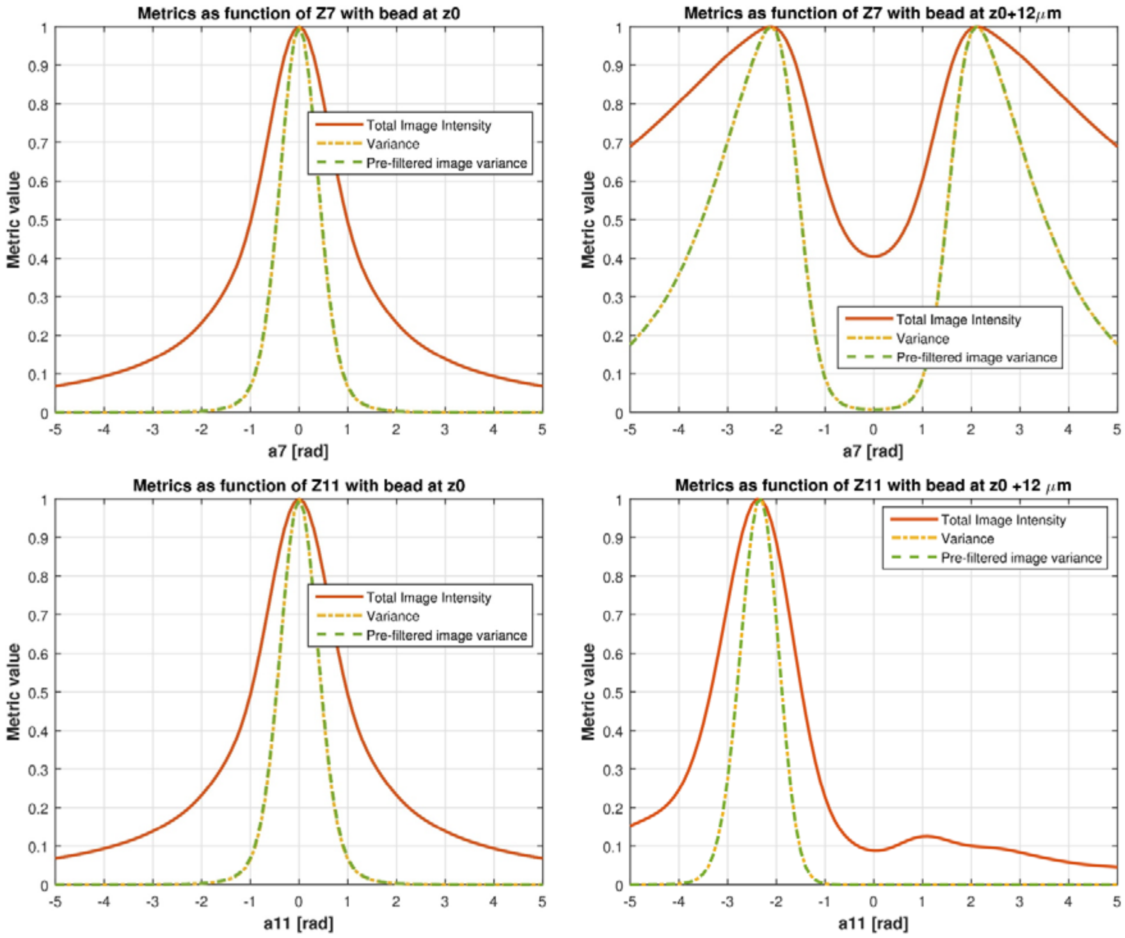

Figure S2: Schematics of the two-photon laser scanning microscope

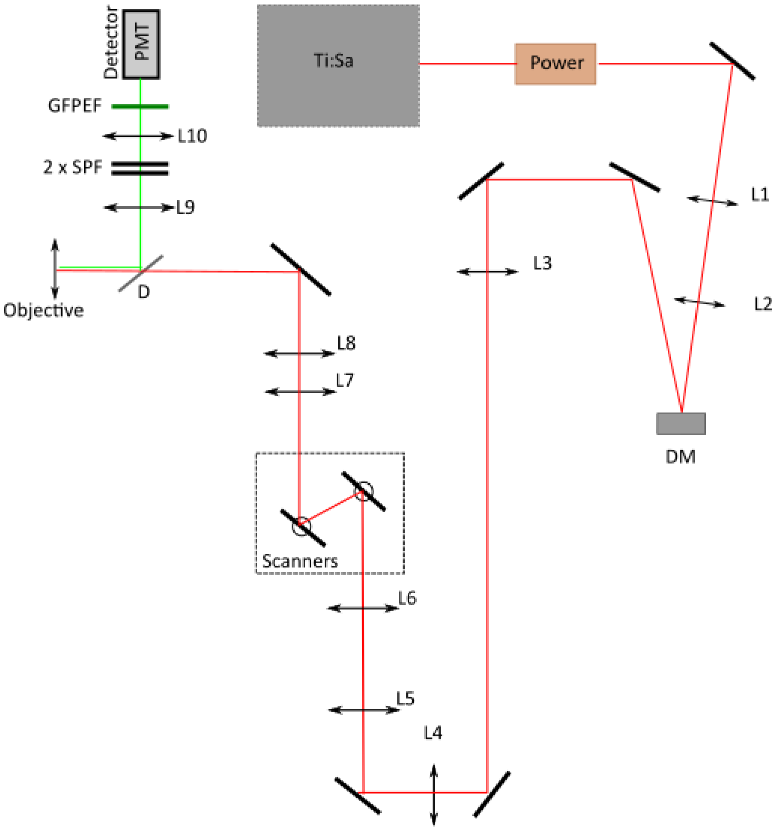

References

Débarre D, Botcherby EJ, Watanabe T, Srinivas S, Booth MJ, Wilson T, Image-based adaptive optics for two-photon microscopy. Opt. Lett. 2009; 34:2495.

Fienup, J. R., & Miller, J. J. Aberration correction by maximizing generalized sharpness metrics. JOSA A, 20(4), 609-620 (2003)

Girkin, J. M. (2013). Implementation of Adaptive Optics in Nonlinear Microscopy for Biological Samples Using Optimization Algorithms. In Kubby, J. A. (Ed.), Adaptive Optics for Biological Imaging (pp. 191-207). CRC press.

Kubby, J. A. (Ed.). (2013). Adaptive Optics for Biological Imaging. CRC press.

Sulai YN, Dubra A. Non-common path aberration correction in an adaptive optics scanning ophthalmoscope. Biomed Opt Express. 2014 Aug 15;5(9):3059-73.

Wahl DJ, Jian Y, Bonora S, Zawadzki RJ, Sarunic MV. Wavefront sensorless adaptive optics fluorescence biomicroscope for in vivo retinal imaging in mice. Biomed Opt Express. 2015 Dec 3;7(1):1-12.
